# Psychometric Evaluation of the Major Depression Inventory (MDI) as a Depression Severity Scale in Chinese Patients With Coronary Artery Disease. Findings From the MEDEA FAR-EAST Study.

**DOI:** 10.3389/fpsyt.2019.00493

**Published:** 2019-07-18

**Authors:** Yixiao Chen, Xiaoyan Fang, Xueqian Shuai, Kurt Fritzsche, Rainer Leonhart, Sophia Hoschar, Li Li, Karl-Heinz Ladwig, Wenlin Ma, Heng Wu

**Affiliations:** ^1^Department of Psychosomatic Medicine, Shanghai Tongji Hospital, Tongji University School of Medicine, Shanghai, China; ^2^Institute of Epidemiology II, Mental Health Research Unit, Helmholtz Zentrum München, German Research Center for Environmental Health, Munich, Germany; ^3^Department of Psychosomatic Medicine and Psychotherapy, Technical University Munich, Munich, Germany; ^4^Department of Psychosomatic Medicine and Psychotherapy, Faculty of Medicine, Medical Center University of Freiburg, Freiburg, Germany; ^5^Institute of Psychology, University of Freiburg, Freiburg, Germany; ^6^College of Electronics and Information Engineering, Tongji University, Shanghai, China; ^7^Department of Cardiology, Shanghai Tongji Hospital, Tongji University School of Medicine, Shanghai, China

**Keywords:** depression, reliability, validity, Major Depression Inventory, acute myocardial infarction

## Abstract

**Background:** It is highly recommended that all patients with coronary artery disease should be screened for depression. The Major Depression Inventory (MDI) is a widely used self-rating scale for the assessment of depression but is not valid in Chinese language. The present study was designed to assess the reliability and validity of a version of the MDI translated into Chinese among patients with acute myocardial infarction (AMI).

**Methods:** Data were derived from the “Multicenter Delay in Patients Experiencing Acute Myocardial Infarction in Shanghai” (MEDEA FAR-EAST) study. Using a cross-sectional study design, the Chinese version of the MDI was administered to a total of 267 inpatients. The internal consistency reliability of the MDI scale was evaluated based on the Cronbach’s coefficient and the binary coefficient for the whole scale. Exploratory factor analysis was performed to assess the internal consistency of the MDI. To examine discriminant validity, we analyzed the correlation of the MDI score with the General Anxiety Disorder-7 (GAD-7) and World Health Organization-5 Well-Being Index (WHO-5) scale scores.

**Results:** The Chinese version of the MDI showed high reliability (Cronbach’s alpha = 0.909, split-half reliability = 0.866). We identified one factor that explained 52% of the variance, which indicated that the MDI has satisfactory structural validity. The correlations of the MDI scores with the GAD-7 scores (*r* = 0.425) and the WHO-5 scores (*r* = −0.365) were moderate, suggesting that the MDI has acceptable discriminant validity.

**Conclusions:** The MDI was proved to be a highly reliable and satisfactory valid diagnostic screening tool to assess depression in Chinese cardiac patients.

## Background

Depression is a seriously disabling public health problem with a very high prevalence (1). The monthly prevalence rate of depression in China is 2.06% (2). However, depression is often undetected for various reasons including somatic symptoms (3). Literature have demonstrated that depression is associated with the onset of somatic diseases such as cardiovascular diseases, diabetes, musculoskeletal diseases, and obesity (4–8). Depression is an independent predictor of cardiovascular mortality in healthy populations (9). Furthermore, in cardiac patients, depression considerably increases the progression of disease as well as the incidence of adverse cardiovascular events and mortality (10). The current number of cardiovascular disease patients in China is 290 million, of which 11 million are coronary heart disease (CHD) (11). These CHD inpatients show higher prevalence of elevated depressive symptoms (30–50%) and major depression (15–20%) than general population (12). Studies have indicated that the major relation between depression and CHD is mainly in the acute states (13). Severe depression in hospitalized patients with acute coronary syndrome can doubled mortality in 6–7 years (14). Moreover, people with major depression disorder are vulnerable to coronary risk factors, such as sedentary lifestyle, smoking, and alcoholism (15, 16). Depression and CHD might share common pathophysiological mechanisms (17). Reviews suggested that assessing depression is needed in the population of CHD patients (18, 19). It is highly recommended that all patients with CHD be screened for depression (20).

However, psychiatric diagnosis of each patient is time and effort consuming. There are some valid depression assessment questionnaires, for example, the Zung Depression Rating Scale (ZRDS) and Beck Depression Inventory (BDI). However, patients with acute coronary artery disease were unable to cooperate with the long diagnostic scale. It is more feasible to use a screening scale that combines diagnosis with concise. The Major Depression Inventory (MDI) is such a brief self-rating scale for the assessment of depression developed by Bech et al. (21) in *Diagnostic and Statistical Manual of Mental Disorder* (4^th^ Edition) (DSM-IV) major depression (22) and in International Statistical Classification of Diseases and Related Health Problems (10^th^ Revision) (ICD-10) moderate to severe depression (23). The MDI has been proved to be useful and valuable with good reliability and validity (24, 25). Moreover, in psychiatric patients, the sensitivity and specificity of the scale are acceptable (26, 27). Therefore, the MDI can easily screen depression. However, the MDI has not been evaluated in China.

The present study was designed to assess the reliability and validity of a version of the MDI translated into Chinese as a depression severity scale among patients with acute myocardial infarction (AMI) and to provide testing and evaluation tools for relevant research on the impact of depression among acute coronary heart disease patients in China.

## Method

### Study Design and Setting

The present data were derived from the data of the “Multicenter Delay in Patients Experiencing Acute Myocardial Infarction in Shanghai” (MEDEA FAR-EAST) study between April 2016 and February 2017. This investigation was replicated from a German study that relied on bedside interviews, self-administered questionnaires, and patient charts to collect biobehavioral, psychological, and baseline patient characteristics. We performed a multicenter cross-sectional observational study at Shanghai Tongji Hospital, Yangpu Hospital, 455 People’s Hospital, and the 10th Hospital. The study was approved by the Ethics Commission of Shanghai Tongji Hospital on 16 March 2016 (伦审-KYSB-2016-74). The approval is applicable for all participating centers (Shanghai Tongji Hospital, Yangpu Hospital, Tenth Hospital, and 455 Hospital). Details have been described in “The MEDEA FAR-EAST Study: Conceptual framework, methods and first findings of a multicenter cross-sectional observational study” (28). This work is a secondary analysis of the MDI score data.

### Patient Recruitment

Participants were consecutively recruited from the population pool of all AMI patients in Shanghai Tongji Hospital together with Yangpu Hospital, 455 People’s Hospital and the 10th Hospital from 11 April 2016. Participation in the study was voluntary. Informed consent was obtained from all participants. When written consent was not possible due to somatic weakness (e.g., poor eyesight), oral consent had to be given and was documented.

The main inclusion criterion was hospitalization with an acute myocardial infarction (AMI), confirmed by typical symptoms at onset such as elevated heart enzymes (troponin I or troponin T) and corresponding ECG diagnosis. There were no age or sex restrictions. The exclusion criteria were prehospital coma/syncope/cardiopulmonary resuscitation that prevented patients from making decisions as well as in-hospital AMI, refusal to participate, and cognitive and language impairment.

**Figure 1:**
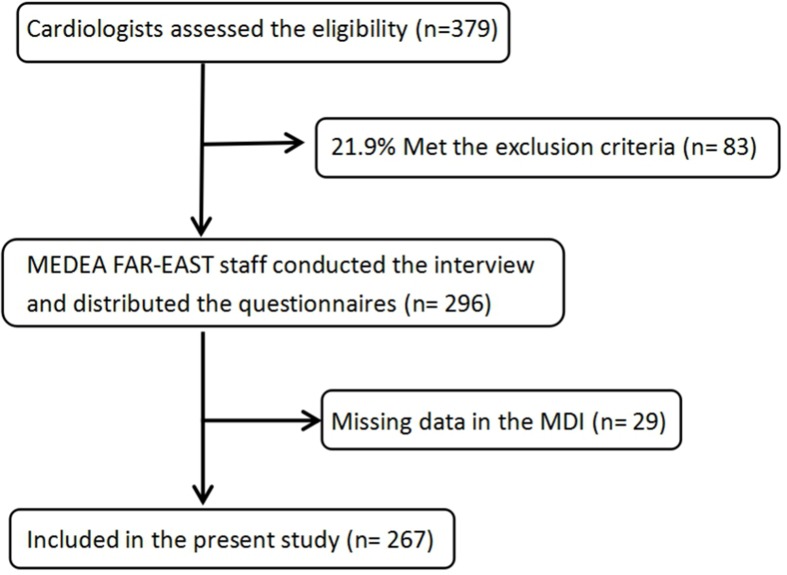
Consort chart of patients in the “Multicenter Delay in Patients Experiencing Acute Myocardial Infarction in Shanghai” (MEDEA FAR-EAST) study.

From mid-April 2016 until mid-January 2017, 379 patients were considered eligible, of which 83 (21.9%) were excluded. The main causes for exclusion were refusal to participate (*n* = 25, 30.1%), language barrier or/and cognitive impairment (*n* = 21, 25.3%), and missing data (*n* = 27, 32.5%). Additionally, 29 patients had missing values on their MDI scores. Thus, the final sample consisted of 267 subjects (see **Figure 1**).

### Assessment Instruments

#### Major Depression Inventory (MDI)

The MDI is a 12-item self-report scale based on the DSM-IV symptoms of major depression and the ICD-10 category of moderate to severe depression. The MDI is a brief self-rating scale for the assessment of depression using the past 2 weeks as the time frame. The MDI contains 12 items, as item 8 and item 10 each have two subitems, a and b. The scoring of each item ranges from 0 to 5 (0 = “at no point of time,” 1 = “now and then,” 2 = “a little less than 1/2 of the time,” 3 = “a little more than 1/2 of the time,” 4 = “most of the time,” 5 = “the whole time”). Major depression was screened according to the ICD-10 diagnosis. Previous studies have reported excellent reliability and acceptable concurrent, criterion, discriminant, and structural validity (24,, 29). The MDI was also reported to have a single-factor structure (21, 24).

#### General Anxiety Disorder (GAD-7)

The GAD-7 was used to measure the severity of generalized anxiety disorders (30). Each of the items is rated on a 4-point Likert scale that ranged from 0 to 3. This instrument showed high internal reliability and good criterion, construct, factorial, and procedural validity (31, 32). In our study, we found a Cronbach’s alpha of 0.911 for the GAD-7.

#### World Health Organization-5 Well-Being Index (WHO-5)

The WHO-5 is a five-item self-report scale that is commonly used to measure subjective psychological and emotional well-being (33). The WHO-5 has also been used as a screening tool for depression (34, 35). Each of the items is rated on a 6-point Likert scale that ranged from 0 to 5. The construct validity of the scale has been evaluated as satisfactory (36). In our study, we found a Cronbach’s alpha of 0.913 for the WHO-5.

#### Interview and Patient Charts

Additionally, we collected the patients’ sociodemographic data and information concerning their general medical conditions *via* interview and patient charts.

### Translation of the Questionnaires

Equivalent language versions of the instruments that assess depression were needed to undertake multicenter research and to obtain meaningful comparisons of the results obtained in different countries. The MDI has been translated into several languages including Arabic, Danish, Dutch, Finnish, French, German, Greek, Swedish, Turkish, Spanish, and Serbian (24, 25, 29), but there has been no validated version available in the Chinese language. Therefore, we translated the MDI and pretested it in 20 patients to ensure that patients were able to understand all items.

The MDI was translated and back-translated from English into Mandarin Chinese using a state-of-the-art procedure for test translation. Following the “ITC-Test Adaptation Guidelines” (Version 2000) of the International Test Commission (ITC) (37), independent translations were completed initially by three native Chinese speakers (a psychiatrist, a psychologist, and an educator) living in the USA, each of whom was fluent in written and spoken English. One of the translators regularly participated in the project meetings. The group discussed the translations and agreed on the version to be moved forward. The Chinese translation was back-translated into English by translators who were blinded to the original version. The working group then compared the English original, the translated Chinese version, and the back-translated English version to create the final version.

The GAD-7 and WHO-5 have been validated and applied in the Chinese language and were published in Chinese research papers (38, 39).

### Statistical Analysis

Using IBM SPSS software (24.0), a single sample was analyzed. For descriptive analyses of the quantitative variables, the mean, standard deviation, and range were calculated, and for analyses of the qualitative variables, the frequencies and percentages were used. The literature has not indicated an official cut-off for MDI scores. Thus, we used the third quartile (MDI score = 18) as a cut-off score to determine prevalence rates, which need further research in new projects. The internal consistency reliability of the MDI scale was evaluated based on Cronbach’s coefficient and split-half reliability. Exploratory factor analysis was used to evaluate the structural validity of the scale. Kendall’s tau-b correlation coefficients were used to assess the correlations of MDI scores with GAD-7 scores and WHO-5 scores. The significance level α was set at 0.05.

## Results

### Study Sample

In the present study, the sample consisted of 267 subjects, which corresponded to a response rate of 90.2%. Of these patients, 82% (*n* = 219) were male, and 18% (*n* = 48) were female. Their ages ranged from 30 to 90 years (mean = 63; SD = 13.2), which was close to a normal distribution.

### Sociodemographic Characteristics

As seen in **Table 1**, the study participants were predominately not living alone, well-educated, and unemployed. Among the participants, 246 (92.1%) were not living alone, 130 (48.7%) had finished high school or a higher level of education, and 171 (64.0%) patients were unemployed.

**Table 1:** Sociodemographic information and clinical characteristics of the 267 patients with acute myocardial infarction.

	Total	Non-MDI (score ≤ 18)	MDI (score > 18)	*P* value
Sample size	267	202	65	
Sociodemographic information				
Age	63 (13.2)	62 (12.5)	64 (15.1)	0.275
Sex (male)	219 (82.0)	168 (83.2)	51 (78.5)	0.390
Sex (female)	48 (18.0)	34 (16.8)	14 (21.5)	
Not living alone	246 (92.1)	188 (93.1)	58 (89.2)	0.371
Education (high school and higher)	230 (86.1)	175 (86.6)	55 (84.6)	0.682
Unemployed	171 (64.0)	123 (60.9)	48 (78.3)	0.058
Somatic risk factors				
Hypertension	162 (60.7)	117 (57.9)	45 (69.2)	0.104
Hypercholesterolemia	61 (23.2)	46 (23.2)	15 (23.1)	0.979
Diabetes mellitus	92 (34.5)	70 (34.7)	22 (33.8)	0.905
Smoking	63 (44.4)	43 (41.7)	20 (51.3)	0.307
Family history	41 (15.4)	28 (13.9)	13 (20.0)	0.232
Obesity	20 (7.6)	15 (7.5)	5 (7.9)	0.909
Previous MI	26 (9.7)	19 (9.4)	7 (10.8)	0.811
Physical inactivity	141 (52.8)	102 (50.5)	39 (60.0)	0.182
Psychological risk factors				
MDI	12 (10.0)	7 (5.3)	27 (6.2)	<0.001
GAD-7	4 (4.1)	3 (3.2)	8 (4.7)	<0.001
WHO-5	64 (23.6)	69 (20.7)	47 (24.9)	<0.001

MI, myocardial infarction; MDI, Major Depression Inventory; GAD-7, General Anxiety Disorder-7; WHO-5, World Health Organization-5 Well-Being Index.

### Clinical Characteristics

As shown in **Table 1**, the most frequently reported cardiovascular risk factors were as follows: hypertension (60.7%), physical inactivity (52.8%), smoking (44.4%), diabetes mellitus (34.5%), hypercholesterolemia (23.2%), family history (15.4%), and obesity (7.6%). A total of 26 patients had previous myocardial infarctions.

### MDI Score

The mean total score on the MDI was 11.93, with a standard deviation of 10.01. The scores did not follow a normal distribution in this population but rather manifested a skewness towards the lower values (median = 9). There were no significant differences in sociodemographic characteristics and somatic risk factors between the two groups. Significant differences in the mean MDI, GAD-7, and WHO-5 scale scores between the MDI and non-MDI groups were found. Patients with higher MDI scores were more likely to suffer from anxiety and suboptimal well-being than those patients with lower MDI scores (see **Table 1**).

### Reliability

Reliability was analyzed in terms of internal consistency using Cronbach’s alpha and split-half reliability for the total scale score. The Cronbach’s alpha was 0.909, and the split-half reliability was 0.866, both of which revealed satisfactory reliability of this scale.

### Factor Analysis

Exploratory factor analysis was used to evaluate the structural validity of the scale. The Kaiser–Meyer–Olkin (KMO) measure of sampling adequacy was 0.919, indicating sample adequacy; Bartlett’s test of sphericity was 1,641.322 (*P* < 0.001), suggesting that the factor analysis was justified in the sample.

By adopting the Kaiser criterion, one factor was extracted, which accounted for 52% of the variance. The eigenvalue of the factor was 6.24. Each item had a common factor load >0.4, which confirmed that the common factor obtained by factor analysis tested the same content (40). This result indicated that the MDI had good structural validity (see **Table 2**).

**Table 2:** Component matrix^a^ of the MDI.

Item	Factor 1
1	0.788
2	0.707
3	0.714
4	0.835
5	0.747
6	0.799
7	0.763
8a	0.766
8b	0.811
9	0.553
10a	0.637
10b	0.418

Extraction method: principal component analysis

a One component was extracted.

### Discriminant Validity

To examine the discriminant validity of the MDI, we used Kendall’s tau-b relevant evaluation to study the relations among the MDI scores, GAD-7 scale scores, and WHO-5 scale scores. The MDI score moderately correlated with the GAD-7 scale score and the WHO-5 scale score. The Kendall’s tau-b correlation coefficient of the MDI scale with the GAD-7 scale score was 0.425 (*P* < 0.001). The correlation coefficients of the MDI scale with the WHO-5 scale score was −0.365 (*P* < 0.001). The MDI score showed a negative correlation with the GAD-7 scale score and a positive correlation with the WHO-5 scale score (see **Table 3**).

**Table 3:** Correlation coefficients between the WHO-5, GAD-7, and MDI.

	1	2
1 MDI		
2 WHO-5	−0.365*	
3 GAD-7	0.425*	−0.291*

*P < 0.01.

## Discussion

Studies have shown an increased association between depression and CHD (1, 13, 14). Our study is the first psychometric evaluation of the Chinese version of the MDI as a depression severity scale in patients with CHD. Participants were selected from AMI patients in hospitals that represented diverse sociodemographic characteristics. Demographic characteristics were comparable to relate studies in China especially in terms of mean age, gender distribution, and educational level (41–43). There are more male than female in the sample, probably because the incidence of AMI in male patients (78.53/l0,000 ∼56.61/l0,000) was higher than that in female patients (50.3l/100,000 ∼31.76/100,000) (11). However, there are no significant differences between the MDI and non-MDI group. The results revealed satisfactory reliability and good validity of the scale. This investigation provides the scientific background and basic requirements for applying the MDI instrument to the Chinese population.

Our finding of a Cronbach’s alpha of 0.909 was indicative of excellent reliability of the items in the Chinese version of the MDI and was consistent with the values reported in previous studies (between 0.89 and 0.94) (26, 27, 29). The binary coefficient for the total scale score was 0.866, which strengthens the evidence of satisfactory reliability.

Furthermore, based on factorial analysis, we identified one factor that explained 52% of the variance, which was consistent with the findings in a previous study. In a factor analysis of a Greek version of the MDI, one factor was identified that explained 54% of the variance (24). A comparative study of the MDI and the ZDRS reported one general factor for the MDI that explained 58.3% of the variance (21).

In addition, evidence for the discriminant validity of the MDI was found, as indicated by the moderate correlations with GAD-7 (0.425) and WHO-5 (−0.365), both of which are specific measures for anxiety and emotional well-being. The results were similar to the correlations of depression with GAD-7 and WHO-5 that were found in other studies. In a study conducted in Turkey in 2010 that included 240 graduate students and 200 outpatients from the Mental Health, Physiotherapy and Rehabilitation, and Cardiology clinic, it was shown that the GAD-7 correlated with the BDI (0.52) and with the Patient Health Questionnaire-9 (PHQ-9) (0.64) (44). The anxiety disorder may interact with depressive disorder in predicting the CHD (45). Patients with depression and comorbid anxiety were 43% more likely to have a subsequent cardiac event compared with either depression or anxiety alone (46). Another study published in 2018, with a sample of 116 Iranian volunteer psychiatric outpatients, reported that the WHO-5 negatively correlated with PHQ-9 (−0.358) and BDI-13 (−0.475) (33).

Moreover, the MDI scores were left skewed. Most of the patients had mild depression, which is either a reflection of the less severe type of depression seen in this population or that patients with severe depression declined to participate. This result was consistent with that of another study in which the BDI-II scores had a positively skewed distribution in cardiac patients (47). However, mild depression in CHD patients still impair functional ability and increase morbidity, mortality, and health care costs (48).

### Strengths and Limitations

This study was investigated in a well-selected homogeneous population. However, it has some possible limitations. Due to the cross-sectional study design, the retest reliability cannot be evaluated. Another potential limitation is that there are no data available to test criterion validity. Thus, we indirectly approached this concept by evaluating the discriminant validity. The MDI scale can discriminate between depression, anxiety, and emotional well-being. Furthermore, the self-report scale may be influenced by the theoretical background of participants.

## Conclusion

Our study translated the MDI into Chinese and proved that the scale has high reliability and satisfactory validity as a depression severity scale for CHD patients. Therefore, we introduced the Chinese version of the MDI as a depression assessment tool for patients with cardiac disease.

## Data Availability

All datasets generated for this study are included in the manuscript and the supplementary files.

## Ethics Statement

The study was approved by the Ethics Commission of Shanghai Tongji Hospital on 16 March 2016 (伦审-KYSB-2016-74). The approval is applicable for all participating centers (Shanghai Tongji Hospital, Yangpu-Hospital, Tenth-Hospital and 455 Hospital).

## Author Contributions

HW, K-HL, KF, and WM contributed to the conception and design of the study; SH organized the database; YC and XS performed the statistical analysis; YC and XF wrote the first draft of the manuscript; HW, KF, and RL wrote sections of the manuscript. All authors contributed to manuscript revision, read and approved the submitted version.

## Funding

This work was supported by the Science and TechnologyCommittee Foundation of Shanghai (16411965500).

## Conflict of Interest Statement

The authors declare that the research was conducted in the absence of any commercial or financial relationships that could be construed as a potential conflict of interest.
